# Surgical outcome of spelenectomy in Thalassemia major in children

**DOI:** 10.12669/pjms.322.8815

**Published:** 2016

**Authors:** Ikramullah Khan Akhtar, Muhammad Ashraf, Irum Uzma Khalid, Mukhtar Hussain

**Affiliations:** 1 Ikramullah Khan Akhtar, Paediatric Surgery Department, Children’s Hospital & Institute of Child Health, Multan, Pakistan; 2 Muhammad Ashraf, Paediatric Surgery Department, Children’s Hospital & Institute of Child Health, Multan, Pakistan; 3 Irum Uzma Khalid, Paediatric Surgery Department, Children’s Hospital & Institute of Child Health, Multan, Pakistan; 4 Mukhtar Hussain, Paediatric Surgery Department, Children’s Hospital & Institute of Child Health, Multan, Pakistan

**Keywords:** Splenectomy, Thalassemia, Haemotological disorder

## Abstract

**Objective:**

To determine the surgical outcome of splenectomy in children with thalassemia major.

**Methods:**

It is an observational and descriptive study conducted in Department of Paediatric Surgery in collaboration with hematology, radiology, anesthesia and paediatric intensive care department at The Children’s Hospital and the Institute of Child Health, Multan during the period of September 2007 to September 2013. A total of 50 patients suffering from thalassemia major already diagnosed and under management reffered from haematology department for splenectomy were included in this study. After admission, patients were assessed on the basis of history, clinical examination, and necessary investigations before surgery and later on follow-up. Investigations carried were CBC, PT, APTT, Viral markers, ECG, X-ray Chest, abdominal ultrasonography and ECHO if necessary. Splenectomy was performed after prophylactic vaccination against post splenectomy infections. Follow up was performed for at least two years. Blood transfusion requirements and number of hospital visits per annum before and after splenectomy were calculated and results analyzed statistically using SPSS-20.

**Results:**

Fifty patients were included in this study. Out of these fifty, 43 (86%) male and 7(14%) were female with a mean age of 9 years. Average blood transfusion requirement was 250 ml/kg/year, interval of blood transfusion was two weeks and twenty five visits per year before splenectomy. After splenectomy, requirement of blood transfusion reduced to 125ml/kg/year, interval between transfusion increased to one month and hospital visits reduced up to twelve per year.

**Conclusion:**

Blood transfusion requirement and number of hospital visits per year are decreased and interval between transfusions is increased after splenectomy. Splenectomy should not be delayed when indicated. Preoperative vaccination decreases the chance of post splenectomy infection.

## INTRODUCTION

Thalassemia major is an inherited haematological disorder leading to anemia in affected children. It is an autosomal recessive trait in which chromosome 11 is involved. It affects synthesis of the Beta globin chain of hemoglobin which is either decreased or absent leading to abnormal shape of Red Blood Cell (RBC). When one of the beta globin chain gene is normal and other abnormal, it is thalassemia minor. If both the genes are involved and disorder presents late in life, it is called as thalassemia intermedia. If both the genes are involved and disorder is manifested early in life, it is called as thalassemia major which is more aggressive disease.^1^

Abnormal shaped RBCs are rapidly destroyed by the reticuloendothelial system particularly spleen leading to microcytic hypochromic anemia, splenomegaly and iron overload. Enlargement of spleen is due to both extra medularry haematopoisis and entrapment of abnormal shape RBC’s. Patient develops pallor in infancy and Protuberant abdomen due to splenomegaly. Skull bossing, maxillary overgrowth, cardio myopathy, and skin pigmentation due to iron deposition may be other clinical features.^1^ Endocrine glands especially pancreas^2^ and pituitary,^3^ may also be involved in iron deposition. Disease is diagnosed by peripheral blood film and serum electrophoresis.^1^

Patient is managed by repeated blood transfusion and iron chelation therapy initially.^4^ Splenectomy is indicated when blood transfusion exceed 250ml/kg/year. Hypersplenism, abdominal discomfort due to massive splenomegaly and splenic injury are other indications of splenectomy in thalassemia patients.^5^ Splenectomy has its own complications such as post splenectomy sepsis, which is manageable with pre-operative vaccination and post-operative use of long acting antibiotics.^1^ This study was carried out with an objective to assess the outcome of splenectomy as a whole as regard to blood transfusion, abdominal discomfort, interval of transfusion, hospital visits and post splenectomy infection.

## METHODS

It is an observational and descriptive study conducted in Department of Paediatric Surgery in collaboration with hematology, radiology, anesthesia and paediatric intensive care department at The Children’s Hospital and the Institute of Child Health, Multan during the period of September 2007 to September 2013. A total of 50 patients suffering from thalassemia major for which splenectomy was performed were included in this study. All these patients were diagnosed and were under treatment in haematology department. After admission the patients were assessed on the basis of history, clinical examination, and necessary investigations before surgery and later on follow-up carried out in department of paediatric surgery.

Necessary investigations were carried out as CBC, PT, APTT, Viral markers, ECG, X-ray Chest, abdominal ultrasonography and Echocardiography if necessary. The parents were counseled about the benefits and complications of splenectomy specially post splenectomy infections. Prophylactic vaccination against post splenectomy infections was given two weeks before surgery. In case of splenic injury, vaccination was done at the time of admission. After complete assessment of patients and consent of parents splenectomy was performed.

Post operative care and management was provided initially in paediatric intensive care unit and later on in paediatric surgical department. Nasogastric tube was placed before surgery and was removed 24 hours after surgery. At the time of discharge, guideline for post splenectomy infection and long acting penicillin therapy was advised. Haemoglobin level was assessed on fortnightly follow up for six months and monthly for another one and half year. Blood transfusion was provided when indicated (haemoglobin< 8 gm). All the information including amount, interval and number of blood transfusion per year before and after was calculated and any complications if occurred after splenectomy were noted. Results were analyzed statistically using SPSS-20.

### Operational Definitions

### Thalassemia Major

It occurs when a child inherits two mutated genes, one from each parent. They lack the ability to produce normal adult hemoglobin. Children born with thalassemia major usually develop the symptoms of severe anemia and spelenomegaly within the first year of life.

### Hypersplenism

Hypersplenism is a condition in which the spleen becomes increasingly active and then rapidly removes the blood cells. It can result from splenomegaly due to any cause but is most common with splenomegaly secondary to haematological disorders as thalassemia major. Rapid fall of haemoglobin level less than 8gm/dl within two weeks interval is a sign of hypersplenism which can only be treated by splenectomy.

### Acute gastric dilatation

It is massive dilatation of the stomach after splenectomy leading to shock. Dilated stomach is filled with gas, secretions and may rotate on its mesenteric axis.

### Overwhelming post splenectomy infection

It is a type of infection which is rapidly fatal occurring in individuals following removal of the spleen caused by encapsulated organisms including Streptococcus pneumonia and typically characterized by either meningitis or generalized sepsis.

### Abdominal discomfort

In thalassemia major hepatosplenomegaly leads to massive abdominal distension causing difficulty in daily routine activity such as going to school and sports.

## RESULTS

Total number of the patients was fifty. Forty-three (86%) male and 7 (14%) female with a ratio of male to female about 6:1. Age of the patients was in between 7-12 years. Splenectomy was performed due to hypersplenism in 38 (76%) patients, abdominal discomfort 9(18%), and splenic injury 3(6%) (Table-I).

**Table-I:** Indication of Splenectomy.

Indication	No. of Patients	Percentage
Hypersplenism	38	76%
Abdominal Discomfort	9	18%
Splenic Injury	3	6%

Before splenectomy interval of blood transfusion was less than two weeks and total volume of blood transfusion were more than 250ml/kg/yr on an average. After splenectomy average interval of blood transfusion was one month and total volume of blood transfusion about 125ml/kg/year in all the patients during first post operative year. After one year it increased upto to 150ml/kg/year in patients of age between 7-8 years, 175ml/kg/year in patients of 9-10 years of age and 200ml/kg/years in patients of 11-12 year (Table-II). Average ferritin level before splenectomy was 4811 ng/ml with chelation therapy on alternate day and one year after splenectomy it was 4214 ng/ml with chelation therapy of three times in a week.

**Table-II:** Average Blood Transfusion Required Before and After Splenectomy.

Age Group	Blood Transfusion Requirement		

	Before Splenectomy	1st year after Splenectomy	2nd year After Splenectomy
7-8	250ml/kg/yr	125ml/kg/year	150ml/kg/year
9-10	260ml/kg/yr	125ml/kg/year	175ml/kg/year
11-12	270ml/kg/yr	125ml/kg/year	200ml/kg/year

Early post-operative hemorrhage occurred in 3(6%) patients, gastric dilatation after removal of naso-gastric tube 2(4%) patients, and post-splenectomy sepsis 2(4%) patients (Table-III).

**Table-III:** Post Splenectomy Complications.

Complication	No. Patients	Percentage
Post-operative hemorrhage	3	6%
Gastric dilatation	2	4%
Post-splenectomy sepsis	2	4%

## DISCUSSION

Fifty patients of Thalassemia major were included in the study, 43 male and 7 female with male to female ratio of about 6:1. It is mentioned in the literature that disease is more common in male.^1^,^2^ Al Saleem AH 2002 mentioned male to female ratio 10:1.^6^ Patients of thalassemia major become symptomatic and diagnosed in the first year of life and splenectomy is performed at the end of first decade of life.^7^ In this study splenectomy was performed during the age of 7-12 years having an average of 9 years, the similar results are reported by Ali S.^8^ and Al Saleem AH 2002 reported the nearer result which was 8 years.^6^

Major indication of splenectomy are hypersplenism, abdominal discomfort and rupture of spleen.^9^ In this study splenectomy due to hypersplenism was performed in 38 (76%) patients, abdominal discomfort 9(18%), and rupture of spleen 3(6%) patients. Similar results were reported by another study conducted by Elmakki.^10^ Splenectomy is advised when blood transfusion requirement becomes more than 250ml/kg per year or interval between blood transfusions becomes less than 2 weeks.^11^ In our study splenectomy was performed on same parameters when interval between blood transfusions was less than two weeks and amount of transfusion more than 250/ml/kg/yr.^7^

According to Lisa Pecorari splenectomy reduces the transfusion requirement to half of the pre splenectomy stage.^5^,^11^ Interval in between blood transfusion becomes double after splenectomy. In this study blood transfusion requirement reduced from 250ml/kg/yr to 125ml/kg/yr and the interval between transfusions increased from two weeks to more than one month initially. In other words number of blood transfusion decreased from 24 to 12 per year. Hathirat mentioned this decrease in the number of blood transfusion from 10 to 5 per year.^12^ As iron chelation therapy is related to destruction of red blood cells, it was also reduced. Average ferritin level before splenectomy was 4811ng/ml with chelation therapy on alternate day and one year after splenectomy it was 4214ng/ml with chelation therapy of three times in a week.

With the passage of time after splenectomy blood transfusion requirement increases again but variably.^6^,^12^ In this study one year after splenectomy, it increased upto to 150ml/kg/year in patients of age between 7-8 years, 175ml/kg/year in patients of 9-10 years of age and 200ml/kg/years in patients from 11-12 years.

Early post-operative hemorrhage occurred in 3(6%) patients within 24 hours of the surgery and 2(4%) were re-explored, and in 1(2%) patient bleeding was managed conservatively. Acute gastric dilatation can be managed by putting naso-gastric tube. In our study, 2(4%) patients developed gastric dilatation after removing naso-gastric tube after 24 hours but managed conservatively by reinserting for twenty-four hours more. Post splenectomy sepsis is a major lethal complication which can be reduced by prophylactic vaccination before splenectomy and long acting antibiotics after splenectomy. In our study 2(4%) patients developed post splenectomy sepsis but with the use of antibiotics were managed conservatively.^13^,^14^ Endocrine complications are mentioned in literature as Ong CK said but no such complications noted in our study and no mortality was recorded.^15^

## CONCLUSION

Splenectomy improved the quality of life in form of school going, sports and decreased in abdominal discomfort. Patient’s health improved after splenectomy because of improvement in haemoglobin level and decrease in blood transfusion requirement. Patient were able to carry out daily activity in a better way and felt less abdominal discomfort and fatigue. Decrease in blood transfusion requirement also reduced the hospital visits and stay. Cost of iron chelation therapy was also reduced. With these changes psychological and financial stress on family was decreased.

## RECOMMENDATION

Splenectomy should not be withheld and performed when indicated. Prophylactic vaccinations and long acting penicillin should be used to decrease the chance of post splenectomy sepsis.
